# An inflammatory pseudotumour of the larynx: a case report and literature review of an unusual tumour

**DOI:** 10.3332/ecancer.2012.273

**Published:** 2012-10-11

**Authors:** Chaido J Dava, Jiannis K Hajiioannou, Anastasios Terzis, John Bizakis

**Affiliations:** 1 Department of Otolaryngology, General University Hospital of Larissa, Viopolis, PC 41110, PO Box 1425, Larissa, Greece; 2 Department of Pathology, University Hospital of Larissa, Larissa, Greece

**Keywords:** *inflammatory myofibroblastic tumour*, *plasma cell granuloma*, *inflammatory pseudotumour*, *laryngeal neoplasms*

## Abstract

**Background:**

Inflammatory pseudotumour (IPT) is a rare benign pseudoneoplastic proliferation of unknown etiology, often showing locally aggressive behaviour. Conflicting theories about exaggerated response to injury versus true neoplastic origin have been suggested.

**Methods:**

We report a case of laryngeal pseudotumour in a 73-year-old man presenting with hoarseness and slowly progressive dyspnea and a short review of the English language literature on the subject.

**Results:**

Management consisted of midline vertical thyrotomy, excision of the tumour, and a temporary tracheotomy. No recurrence observed eight months postoperatively.

**Conclusions:**

Laryngeal IPT is extremely rare, and it may easily be misinterpreted as a malignant tumour. Conservative excision and anti-inflammatory therapy are advocated, since its general behaviour is benign.

## Introduction

Inflammatory pseudotumour (IPT) is a rare lesion composed of exuberant myofibroblastic proliferation and an inflammatory component. IPT is a controversial entity. Although it is histologically benign, it often shows locally aggressive behaviour that mimics a neoplastic process. Although lungs and orbits are the most common sites of involvement, it has been reported in virtually any tissue or organ of the body [1–3]. The presence of this lesion in the larynx is rare [3–5], and only a few additional cases have been reported since it was first described by Wenig *et al *in 1992 [6]. Due to its rarity, the conception of IPT altered with time from a benign reactive process to an intermediate neoplasm. The hallmarks of IPT are solid nodular growth pattern and abundant myofibroblasts, which have been demonstrated by immunohistochemistry and ultra-structural studies [6]. The purpose of this article is to present a new case of laryngeal IPT in a 73-year-old man situated on the left true vocal cord extending in the immediate subglottic area and discuss its diagnostic and therapeutic aspects. Moreover, a brief review of the English language literature is performed.

## Case report

**Figure 1:**
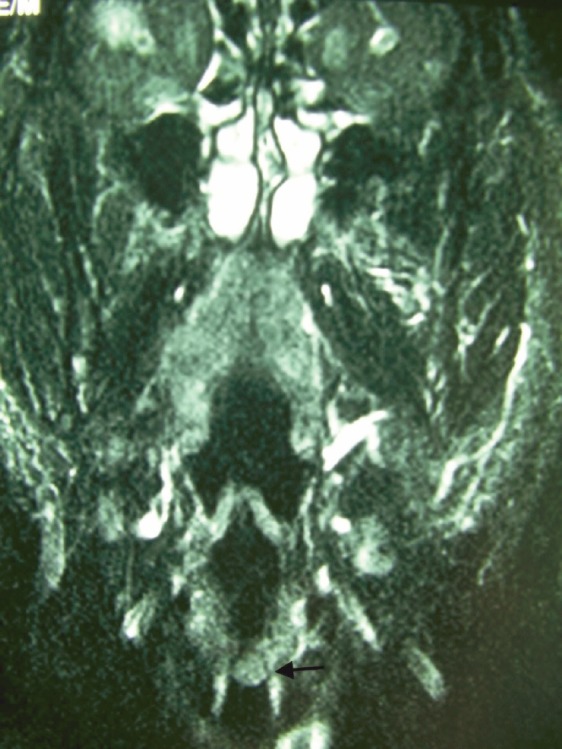
Coronal T1-weighted MRI reveals thickening of the left vocal cord (black arrow).

**Figure 2:**
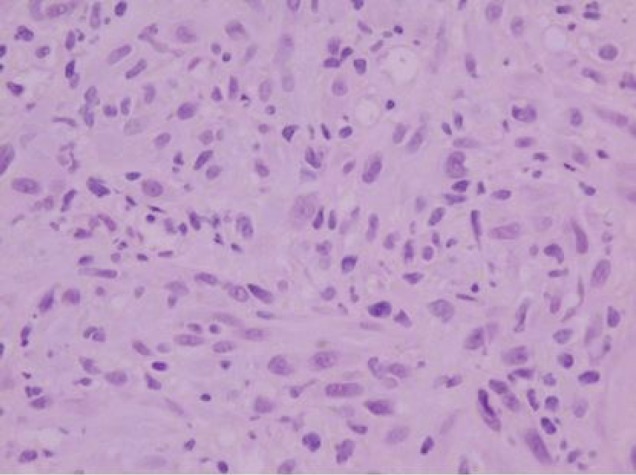
Histologically, the tumor is composed of spindle-shaped myofibroblasts in collagenous and inflammatory background (hematoxylin-eosin stain, original magnification ×400).

**Figure 3:**
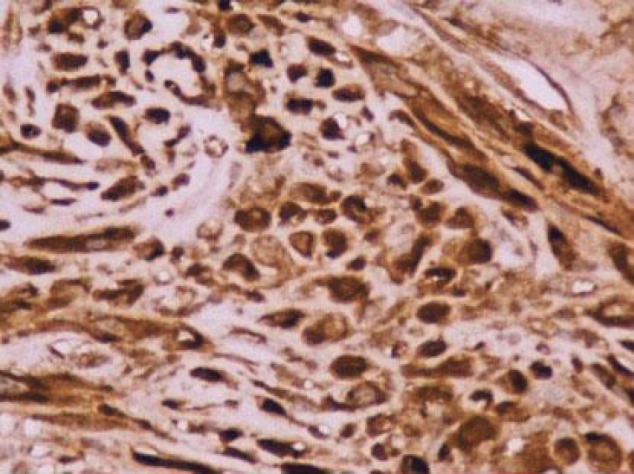
Reactivity in the cytoplasm of the myofibroblasts with smooth muscle actin (original magnification ×400).

**Figure 4:**
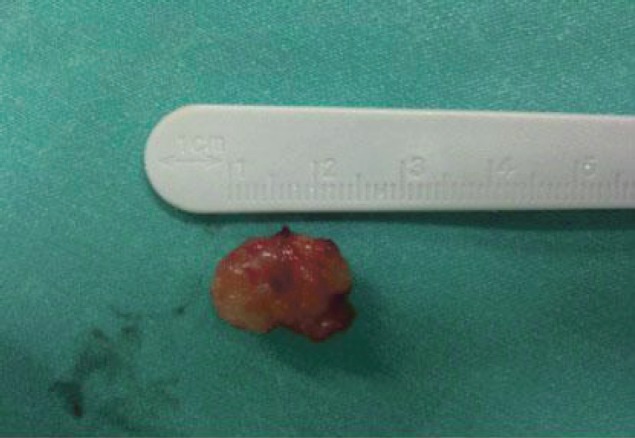
Grossly, the tumor was firmly attached to the left true vocal cord and presented to be nodular, measuring in diameter 1.7 × 1.1 × 0.7 cm.

A 73-year-old man was referred to the ENT Department of the University Hospital of Larissa complaining of persistent hoarseness and slowly progressive dyspnea during the past year. The patient had undergone microlaryngoscopy with biopsies for a left vocal cord tumour nine years ago and his histology examination revealed hyperplasia of the surface epithelium, focal keratosis, slight chronic inflammation, and subepithelial scarring. He was a smoker with medical history of chronic renal failure, coronary artery disease, and diabetes. Endoscopic examination revealed a large nodular lesion along the left true vocal cord with impaired laryngeal mobility ipsilaterally. Chest X-ray was normal. MRI scan showed soft tissue fullness in the area of the left vocal cord (Figure 1). No lymph node enlargement was found in the neck, while the rest ENT examination was unremarkable. Microlaryngoscopy under general anaesthesia confirmed the existence of the lesion on the left true vocal cord and the extent of it in the immediate subglottic area. Biopsy specimens were taken and on histological examination the patient diagnosed as having IPT; abundant myofibroblasts with scattered inflammatory cells were seen, cellular pleomorphism was demonstrated, and mitoses were absent. Immunohistochemically stromal cells were positive for vimentin, SMA, and calponine; CD34 and S100 were focally positive, while ALK1 staining and desmine resulted in negative reactions (Figures 2 and 3). The patient subsequently underwent a median vertical thyrotomy under general anaesthesia, after the airway was secured by a tracheotomy at the beginning of the procedure. The lesion was excised including some surrounding muscle fibres, which appeared to be involved. Grossly, the tumour was firmly attached to the left true vocal cord and presented to be nodular, measuring in diameter 1.7 × 1.1 × 0.7 cm (Figure 4). Prophylactic perioperative antibiotics were administered preoperatively and continued for five days postoperatively. The patient was decanulated on the third postoperative day and had an uneventful recovery. Two months postoperatively, the left true vocal cord had regained mobility and his voice had improved enough. No recurrence has been noticed eight months postoperatively.

## Discussion

Inflammatory pseudotumour (IPT) is an unusual benign solid mass composed of myofibroblastic spindle cells with acute and chronic inflammatory cells that mimics a neoplastic process [1, 3, 7]. To date, 39 laryngeal IPT have been described in the English literature. The mean age of these patients is 44.5 years (range 2–74 years) and the male:female ratio is 1.6:1 [5, 6, 8–10]. Several terms like plasma cell granuloma, histiocytoma, plasma cell histiocytoma complex, fibrohistiocytoma, hamartoma (lymphoid, myxoid), xanthomatous granuloma, inflammatory fibrosarcoma, and benign myofibroblastoma have been used [2, 4–7]. In 1994, World Health Organization (WHO) had anthologized those terms as inflammatory myofibroblastic (IMT) [7]. The IPT was first reported in the lung by Brunn in 1939 [3], whereas a laryngeal occurrence of IPT was first described in 1992 [1, 2, 6, 11]. The upper respiratory tract accounts for 11% of extra-pulmonary cases of IPT and the remainder of the head and neck sites less than 5% [1, 7–9]. IPT must be differentiated from a variety of benign and malignant soft tissue tumours like anaplastic large cell lymphoma, low-grade fibrosarcoma, rhabdomyosarcoma, malignant peripheral nerve sheath tumour, leiyomyosarcoma, spindle cell sarcoma, heamangiopericytoma, plasma cell neoplasm, inflammatory fibroid polypus, chronic fungal infection, Wegener’s granulomatosis, etc [2–4, 7, 10]. Differential diagnosis of glottic growth in children 8–10 years old should include recurrent respiratory papillomatosis, vocal polyp, and granulomatous pathology [3, 9, 11]. 

The origin of IPT is still disputed [6, 7]. One of the theories suggests an association with trauma. The composite cells of IPT are of those that would be expected at the site of wound healing, advocating a reactive-inflammatory origin [2, 5, 6, 9, 10, 12]. In the first cases reported by Wenig *et al*, no history of trauma was present except in one patient who had a traumatic intubation for a totally unrelated procedure [6]. Furthermore, the potential subclinical traumatic stimuli like voice abuse, excessive coughing, and acid-reflux are often associated with wound healing which includes the presence of myofibroblasts. In certain instances, this reaction can be so proliferative resulting in the presence of a clinically apparent lesion dominated by the presence of myofibroblasts [2, 5, 6]. Another theory includes the presence of a trigger such as smoking, as at least half of the reported patients with laryngeal IPT were smokers [6]. Association with the alcohol has not been established in anyone of the reported cases. Epstein–Barr virus has been also associated with IPT at other locations except from larynx [5, 6]. Two studies from Spain have reported the presence of HHV8 transcripts in IPT and suggested a possible link to its pathogenesis [6]. Recently, IPT has been considered a type of IgG4-related sclerosing disease, which includes autoimmune pancreatitis and sclerosing sialadenitis [5, 6, 8]. The concept of IPT being an exuberant post-inflammatory reaction is also supported by the successful application of anti-inflammatory drugs in therapy [5, 6, 10].

The neoplastic theory has been supported by the occasional recurrence and even metastatic potential of IPT (not reported for laryngeal localization, up to day). Aneuploidy, clonality, chromosomal rearrangement at chromosome 2p23, expression of ALK1 [5], p80 [5–7, 10], and loss of eterozygosity have also been demonstrated [6]. Overexpression of ALK protein, as well as the proliferation marker Ki-67 and the apoptosis protein inhibitor Bcl-2, has been correlated with tumorigenesis [5, 7]. Moreover, the molecular evidence by CGH (Comparative Genomic Hybridization) of a loss on chromosome 13p14–22, which is the location of the tumour-relevant retinoblastoma gene (Rb), was first reported by Volker *et al *who presented a 34-year-old woman with IPT and supported the hypothesis of a true neoplastic process [7]. Changes on chromosome 13p are considered to be a common feature in many types of cancer [6].

Clinical characteristics suggestive of IPT include patients in their 30s–40s, hoarseness of varying severity and duration [6, 7], persistent low-grade fever, leukocytosis, lymphocytophilia, neutrophilia, increased erythrocyte sedimentation rate without preceding contagious infection contacts. Under videolaryngoscopy, a solid firm pedunculated submucosal mass (polypoidal or nodular) involving the vocal cords is usually seen in combination with a traumatic history and initial response to steroid therapy without total regression. On imaging, IPT appears to be a well-enhanced elongated mass on the contrast-enhanced CT scan [7]. On MRI, it presents as a well-enhanced lesion on gadolinium-enhanced T1-weighted images, while it shows mixed high- and low-signal intensity on T2-weighted images [1, 2]. If two of the above clinical characteristics are fulfilled in combination with the radiological findings, IPT should be suspected, unless no spindle cell is seen on histological examination [5–11]. Its histopathologic nature is benign and characterized by spindle or satellite cells distributed in a chronic inflammatory background and often displaying a storiform, nodular, or fascicular proliferation pattern. The nuclei of these cells are rounded to oval and display a low-grade pleomorphism within the benign range. These cells are suspended in a myxoid, fibrous, or fibrillary stroma [6, 13]. Batsakis *et al *considered two different types of lesion within this generic term: inflammatory/reparative and myofibroblastic proliferations [13]. According to Coffin’s classification, there are three patterns IPT which may coexist in different areas of the same lesion: (1) hypocellular pattern (myofibroblast organized loosely within edematous myxoid background with plasma cells, lymphocytes, eosinophils, and blood vessels), (2) hypercellular pattern (densely aggregated elongated, short spindle cells, or stellate cells arrayed in collagenized background), and (3) collagen sheets pattern (sparsely cellular component resembling scar tissue) [2, 4, 7]. Some other histological features are absence of necrosis, a mitotic rate of less than two per 10 high-power fields, absence of atypical figures of mitosis, absence of infiltrating growth, mild cellular pleomorphism and presence of polyclonality of plasma and mixed inflammatory infiltrate mostly consists of plasma cells. Lymphocytes, eosinophils, and histiocytes are also present. The overlying mucosa can be inflamed, ulcerated, or hyperplastic, but no dysplasia has been reported. Variable vascularity has been seen. The blood vessels may be scarce or abundant. Ultra-structured studies show spindle cells with elongated cytoplasmic processes [6–10, 13]. Furthermore, Wenig *et al *in their initial paper, where they presented the first series of eight laryngeal IPT, mostly described them as being unencapsulated mass [6, 10].

The immunohistochemical panel is of great assistance in the diagnosis of laryngeal IPTs [7]. The stromal cells in IPT are usually positive for muscle-specific actin, vimentin, and smooth muscle actin (SMA) [6, 10]. Rarely, it is stained positive with desmin and cytokeratins [6, 9]. According to Ong *et al*, IPT is stained negative with h-cladesmon, S-100, and CD34 [7]. Also, Coffin *et al* first demonstrated the ALK1 expression immunohistochemically (36%) and ALK gene rearrangements by fluorescence in situ hybridization (47%) [6]. Immunoactivity of ALK is considered to be significant mostly in IPT of patients younger than 40 years. ALK1 negative cases could represent the IgG4+ variant [5, 7].

Laryngeal IPT should be differentiated from malignant neoplasms to avoid unnecessary overtreatment [4, 6]. Surgical excision with free margins is the treatment of choice [7, 13]. The endoscopic excision with or without laser is considered to be the first line of treatment [9, 14]. This may be followed by high-dose steroids for 6–12 weeks. Steroids may be introduced 2–3 days prior to surgery to reduce peripheral inflammation facilitating the resection [2]. If excision would result in significant function loss, a course of steroids alone may be attempted. Three cases of laryngeal IPT reported in literature treated by prednisolone alone did not show recurrence. Open excision should be reserved for cases of recurrence, poor endoscopic visualization prohibiting complete excision of the mass or when malignancy cannot be excluded [5, 9, 14]. Radiation therapy can be successful in cases of local recurrence [9, 10]. Chemotherapy has been reported for a few cases of recurrent IPT or malignant transformation [2]. Steroid or radiation therapy as sole treatment seems less effective [6, 10]. Focal gross positive margin can be treated by Gamma-Knife/CyberKnife Stereotactic Radio Surgery after surgical debulking [7].

The recurrence rates range from 18 to 21% [5, 9]. The risk of relapse in lesions with positive margins is always higher compared to those with negative surgical margins, despite the aid of post-operative adjuvant therapy [5, 7, 9]. Also, the risk of sarcoma transformation is high in cases of IPT with a history of multiple surgical resections within a short period (nine months) [7] and in cases of inconsistent radiotherapy treatments [3, 7].

Due to the recurrence potential as shown by the case reported by Corsi *et al*, and by one of the cases of Wenig *et al*, a diagnosis of IPT should alert the clinician to strict follow-up affected patients, because there are no morphological features that can predict its biological behaviour [4, 10, 13]. Patients should be followed up every 1–2 months for the first two years; every 2–3 months for the third year and twice a year for the fourth and fifth year. Enhanced CT and/or MRI scan of the neck targeted on the larynx and chest CT (or chest X-ray) are recommended every six months or when required. In patients with gross residual disease, CT or MRI should be ordered 1-month postoperatively [7].

In conclusion, laryngeal IPT is a rare benign pseudoneoplastic proliferation that should be differentiated from malignant laryngeal tumours, both due to its reduced aggressiveness and its respectability by conservative surgery, unlike neoplasms. For achieving optimal results, the surgeon should balance between preservation of function and completeness of excision. Strict follow-up is required due to its potential for recurrence.

## Summary

- Inflammatory pseudotumour (IPT) is a stromal tumour with low aggressiveness.
- Laryngeal inflammatory pseudotumour is a rare benign pseudoneoplastic proliferation, which should be differentiated from malignant laryngeal tumours.
- Surgical resection with negative margins remains the gold standard treatment for laryngeal IPT.
- The surgeon should balance between preservation of function and completeness of excision.
- Strict follow-up is required due to its potential for recurrence.
